# Erfolgsfaktoren und Hindernisse bei der Implementierung kompetenzorientierter Lehre in der Chirurgie

**DOI:** 10.1007/s00104-024-02107-9

**Published:** 2024-06-03

**Authors:** C. Kruppa, M. Rudzki, D. J. Baron, M. Dudda, T. A. Schildhauer, S. Herbstreit

**Affiliations:** 1 Chirurgische Universitätsklinik und Poliklinik, Berufsgenossenschaftliches Universitätsklinikum Bergmannsheil, Ruhr-Universität Bochum, Bochum, Deutschland; 2 Zentrum für Muskuloskelettale Chirurgie, Universitätsmedizin Essen, Universität Duisburg-Essen, Essen, Deutschland; 3 Klinik für Orthopädie und Unfallchirurgie, BG-Klinikum Duisburg, Universität Duisburg-Essen, Duisburg, Deutschland; 4 Institut für Didaktik in der Medizin, Medizinische Fakultät, Universität Duisburg-Essen, Essen, Deutschland

**Keywords:** Kompetenzorientierung, Masterplan, NKLM, NKLC, Unterricht am Krankenbett, Blockpraktikum, Competence-oriented lessons, Master plan, NKLM, NKLC, Bedside teaching, Block internship

## Abstract

**Hintergrund und Fragestellung:**

Für die kompetenzorientierte Ausrichtung der Lehre an den Fakultäten gilt es, die beeinflussenden Faktoren zu identifizieren, um Stärken zu nutzen und Schwächen auszugleichen. Die vorliegende Arbeit untersucht die Erfolgsfaktoren und Hindernisse bei der Implementierung kompetenzorientierter Lehre in der Chirurgie aus dem Blickwinkel der Studierenden und Dozierenden.

**Methoden:**

Nach Implementierung kompetenzorientierter Lehre, basierend auf den Lernzielen des NKLM, in den Kursen Unterricht am Krankenbett Chirurgie (UAK) und Blockpraktikum Chirurgie (BP) an zwei Kliniken wurden Fokusgruppeninterviews und Fragebogenerhebung mit Studierenden (S) und Dozierenden (D) mit anschließender qualitativer Inhaltsanalyse mit Quantifizierung der Aussagen durchgeführt.

**Ergebnisse:**

Im SoSe 2022 nahmen 31 Studierende und 14 Dozierende an Fokusgruppeninterviews teil. 143 Fragebögen (123 S, 20 D) wurden ausgewertet. Erfolgsfaktoren waren für die Studierenden das Vorhandensein konkreter Lernziele/Kompetenzen mit den Aspekten eines „Leitfadens für den Unterricht“, „Transparenz der Lernziele“ und „Einforderung möglich“ sowie „Unterrichtsablauf/-struktur“ und „Betreuung“; das Vorhandensein konkreter Lernziele/Kompetenzen mit den Aspekten „Hilfestellung zur Vorbereitung“ und „Strukturierung des Unterrichts“ sowie das Engagement der Studierenden stellten für die Dozierenden die Erfolgsfaktoren dar. Die Fragebogenerhebung ergab, dass die Mehrheit (88 % S, 75 % D) über die Lernziele informiert war und diese als verfolgt ansahen (84 % S, 95 % D). Als Hindernisse wurden die Faktoren „Zeit“, „Betreuung“ und „Information“ genannt. Faktoren, die nicht eindeutig positiv oder negativ zuzuordnen waren (indifferente Einflussfaktoren), stellten u. a. „Unklarheit, was Kompetenzorientierung ist“ und „Unsicherheit, wie diese zu überprüfen ist“ dar.

**Diskussion:**

Die klare Strukturierung, transparente Lernziele und funktionierende Betreuung sind die Erfolgsfaktoren für die Implementierung und sollten als Stärken genutzt werden. Indifferente Faktoren sind als Hindernisse zu werten und u. a. durch Schulung zu lösen. Die immanenten Probleme des Zeit- und Personalmangels behindern auch diese Implementierung und bedürfen genereller struktureller Veränderungen.

**Zusatzmaterial online:**

Zusätzliche Informationen sind in der Online-Version dieses Artikels (10.1007/s00104-024-02107-9) enthalten.

## Abstract (en)

**Background and objectives:**

For successful competence-oriented teaching at the medical faculties it is important to identify the factors that influence its implementation in order to benefit from the strengths and balance out weaknesses. The present study examined the success factors and obstacles of the implementation of competence-oriented teaching in the surgical discipline from the point of view of students and lecturers.

**Methods:**

After implementation of competence-oriented teaching based on the teaching goals of the NKLM, in clinical examination courses (bedside teaching and block internship, BP) at two hospitals, a qualitative content analysis and quantification of the answers were performed using focus group interviews and questionnaires with students (S) and lecturers (D).

**Results:**

During the summer semester 2022 a total of 31 students and 14 lecturers were interviewed in focus groups and 143 questionnaires (123 S, 20 D) were analyzed. For the students the presence of concrete competences/teaching goals, guidelines for the lesson, transparent goals and ability to demand teaching goals as well as structured lessons and mentoring were the main success factors. Lecturers on the other hand reported the presence of concrete goals, assistance for the lesson preparation and the activity of the students as success factors. The results of the questionnaires showed that the majority (88% S, 75% D) were informed about the teaching goals and considered them to be followed (84%S, 95% D). Obstacles were the factors “time”, “mentoring” and “information”. Factors that were between negative and positive (indifferent factors) were “uncertainty about competence-orientation” and “uncertainty how to examine the teaching success”.

**Discussion:**

Transparent structure and teaching goals as well as a mentoring system are the success factors for the implementation of competence-oriented lessons and should be used as strengths. Indifferent factors represent weaknesses and need to be addressed by training and instruction. Restricted time and personnel resources are the immanent problems that hamper the implementation and require fulminant structural changes.

## Hintergrund und Fragestellung

In den vergangenen Jahren ist die kompetenzorientierte Ausrichtung im Medizinstudium vorangeschritten und hat, unter anderem durch die Betonung durch den Wissenschaftsrat 2014, auch in Deutschland eine zunehmende Bedeutung erlangt [26]. Auch für die chirurgischen Disziplinen ist daher eine zukunftsorientierte Lehre mit Fokussierung auf die Lernenden notwendig [12]. Als Orientierung dient der Nationale Kompetenzorientierte Lernzielkatalog Medizin (NKLM) in der aktuellen Version 2.0 des Medizinischen Fakultätentags (MFT; [6–8]). Ziel des NKLM ist die Umsetzung eines Kernkurrikulums zur Vermittlung ärztlicher Kompetenzen in der studentischen Ausbildung. Durch ihn soll das Kernkurrikulum an neue wissenschaftliche sowie klinisch-praktische Erfordernisse angepasst, eine Vergleichbarkeit der Ausbildungsqualität gewährleistet und auf die ärztliche Weiterbildung vorbereitet werden [7]. Der NKLM wird in Zukunft als verpflichtende Grundlage des Kernkurrikulums im Medizinstudium erwartet. Damit kommt es zu einem Paradigmenwechsel von der fachbasierten Vermittlung von Wissensinhalten zu einer stärkeren Kompetenzorientierung in der Medizinerausbildung. Weiterhin sollen die Verantwortlichkeit für die zu erreichenden Kompetenzen und Lernziele auf studentischer Seite gefördert, das selbstregulierte Lernen unterstützt und die Studierenden auf die Praxis vorbereitet werden [25].

Die Herausforderungen bei der Umsetzung strukturierter klinisch-praktischer Lehre in Fächern mit PatientInnenversorgung sind vielfältig. Multifaktoriell bedingte personelle Engpässe erschweren die beständige Betreuung. Zeit- und Personalmangel, eine zu hohe Anzahl an Studierenden und zu geringe didaktische Vorkenntnisse werden fakultätsübergreifend von Dozierenden beklagt [13]. Möglicherweise kann die Kompetenzorientierung in der Lehre durch klare Fokussierung auf Lehr‑/Lernziele und begleitende Materialien dem entgegenwirken. In einer Befragung von Dozierenden konnte gezeigt werden, dass der Wunsch nach differenzierteren Vorgaben zu Inhalt und Aufbau einer Lehrveranstaltung besteht [13]. Die Aufgabe der Fakultäten ist es, Wege zu entwickeln, die Kompetenzorientierung in der Lehre umzusetzen. Neue Formate befinden sich derzeit in Erprobung und verschiedene Arbeiten über die Evaluation solch kompetenzbasierter Methoden wurden bereits publiziert [2, 4, 25]; sowohl mit Bezug zu praktischen Prüfungen [20, 21] als auch bei der Vermittlung einzelner praktischer Fertigkeiten [10, 17]. Darüber hinaus gibt es bereits Erfahrungen mit einem fragen- und reflexionsbasierten Portfolio im chirurgischen Blockpraktikum [9]. Bei der Implementierung einer kompetenzorientierten Studiengangsentwicklung in der Humanmedizin wird insbesondere die Bedarfs- und Bedingungsanalyse bei den Beteiligten als wesentlicher Schritt empfohlen [19]. Bei der Implementierung kompetenzorientierter Lehre in der Chirurgie gilt es also zu klären, wie dieses erfolgreich gelingen kann und was dabei hinderlich wirkt.

Mit unserer Arbeit sollen erstmals, nach Entwicklung des chirurgischen Unterrichts mit dem Ziel der Kompetenzorientierung, Erfolgsfaktoren und Hindernisse bei der Implementierung, aus Sicht der Studierenden und der Dozierenden, identifiziert werden. Durch Nutzung der identifizierten Erfolgsfaktoren und Minderung der identifizierten Hindernisse können zukünftige Anpassungen unterstützt werden. Durch Einschluss von Studierenden und Dozierenden der Ruhr-Universität Bochum sowie der Universität Duisburg-Essen sollen fakultätsübergreifende Sichtweisen mit einfließen.

## Studiendesign und Untersuchungsmethoden

### Implementierung

Am BG-Universitätsklinikum Bergmannsheil Bochum erfolgte die kompetenzorientierte Veränderung der Lehreinheiten mithilfe eines den Unterricht am Krankenbett Chirurgie (UAK) und das Blockpraktikum Chirurgie (BP) begleitenden Manuals, basierend auf den Lehr‑/Lernzielen des NKLM. Am Universitätsklinikum Essen wurde die kompetenzorientierte Lehre im Blockpraktikum Chirurgie mithilfe eines von Studierenden geführten Portfolios implementiert. Hierbei erfolgte jeweils die Kompetenzorientierung des Unterrichts durch die Umgestaltung der Lehre anhand transparenter Lernziele mit Berücksichtigung der unterschiedlichen Kompetenzebenen. Die Dozierenden wurden über die Veränderung der Lehreinheiten vorab informiert und in die Veränderung eingewiesen. Die Information wurde seitens der Dozierenden an die Studierenden weitergeben. Den Studierenden und den Dozierenden standen sowohl das Manual als auch das Portfolio vor, während und nach der Unterrichtseinheit per Moodle sowie im klinikinternen Intranet und als Ausdruck zur Verfügung. Nach erfolgter Implementierung wurden die Wahrnehmung und der Umgang mit dieser analysiert.

### Studiendesign

Als Forschungsmethode zur Erfassung und Darstellung von Erfolgsfaktoren und Hindernissen bei der Implementation kompetenzorientierter Lehre wurden im Sommersemester 2022 Fragebogenerhebungen und Fokusgruppeninterviews mit Studierenden und Dozierenden beider Fakultäten zur deskriptiven Analyse und qualitativen Inhaltsanalyse gewählt. Diese Methoden eignen sich insbesondere zur Darstellung der Wahrnehmung und Erfahrungen mit Interventionen in der Medizinausbildung [23]. Für die Teilnahme an den Fokusgruppeninterviews waren bei den Studierenden mindestens drei absolvierte Termine UAK oder entsprechend das 2‑wöchige Blockpraktikum in den genannten Kliniken Voraussetzung. Angesprochen wurden alle Studierenden, die diese Voraussetzungen erfüllten. Eine spezifische Selektion erfolgte dabei nicht. Die Dozierenden mussten entsprechende Lehreinheiten unterrichtet haben. Auch hier wurden alle Dozierenden ohne weitergehende Selektion angesprochen. Ausgeschlossen wurden Studierende und Dozierende, die nicht in den entsprechenden Lehrformaten teilgenommen hatten oder die freiwillige Teilnahme ablehnten. Ein positives Ethikvotum sowie eine Datenschutzerklärung und Einverständniserklärung der Teilnehmenden lagen vor Erhebung der Daten vor.

### Fragebogenerhebung

Mithilfe einer Fragebogenerhebung wurden Studierende und Dozierende zu den Lehr‑/Lernzielen, der Vermittlung sowie zu konkreten Kompetenzen im Rahmen des Unterrichts am Ende eines jeden Unterrichtsblocks befragt. Alle 132 teilnehmenden Studierenden wurden gebeten, an der Erhebung freiwillig teilzunehmen. Die Kompetenzvermittlung wurde hier für die Bereiche „Anamneseerhebung“, „klinische Untersuchung“ und „Entwicklung eines Behandlungsplans“ unterschieden. Darüber hinaus wurde bez. der Vermittlung theoretischen Wissens und praktischer Skills, unter Berücksichtigung unterschiedlicher Kompetenzebenen, um Einschätzung gebeten. Diese wurden durch die Verwendung entsprechender Formulierungen bei der Abfrage unterschieden: „theoretisches Wissen vermittelt bekommen“ (Ebene 1), „gesehen und demonstriert bekommen“ (Ebene 2), „selbstständig angewendet haben“ (Ebene 3a), „routiniert ohne Supervision anwenden können“ (Ebene 3b). Die Studierenden und Dozierenden erhielten für sie angepasste Fragebögen (Supplement 1). Die anonyme Beantwortung war in Form einer 6‑teiligen Likert-Skala möglich. Die statistische Auswertung erfolgte mithilfe von Excel (Microsoft Office 2019) und SPSS (IBM, 28.0).

### Fokusgruppeninterviews

Zur Durchführung der Fokusgruppeninterviews erfolgte die Entwicklung literatur- und erfahrungsbasierter Interviewleitfäden (Supplement 2). Im Rahmen der Interviews wurden die wahrgenommenen Erfolgsfaktoren und Hindernisse erfragt sowie die Nutzung des Manuals/Portfolios und dessen Beeinflussung des Unterrichts durch diese diskutiert. Ferner erfolgte ein Austausch über das Wissen und Ziele zu Kompetenzorientierung sowie die erlebte Angemessenheit der abverlangten Kompetenzebenen. Studierende und Dozierende wurden getrennt voneinander interviewt. Demographische Daten wie Alter, Geschlecht und Vorerfahrung in der Chirurgie bzw. Lehrerfahrung wurden erhoben. Die Interviews fanden entweder in Präsenz oder im Rahmen einer Videokonferenz statt. Alle Interviews wurden anonymisiert als Audiodatei gespeichert. Für die qualitative Inhaltsanalyse (nach Kuckartz [14, 16]) erfolgte zunächst die deduktive Kategorienbildung durch die gestellten Fragen. Aus dem erhobenen Material erfolgte anschließend eine weitere induktive Kategorienbildung (Supplement 3). Im Rahmen der Auswertung konnten nicht alle Aussagen eindeutig den positiven (Erfolgsfaktoren) oder negativen Einflussfaktoren (Hindernissen) zugeordnet werden. Um eine Fehlinterpretation zu vermeiden, entschieden sich die Autoren für die Erweiterung um eine dritte Kategorie „indifferente Einflussfaktoren“. Während des Codierungsprozess wurden mehrere Kategorien und Codes induktiv „in Abhängigkeit der Kompetenzorientierung“ erstellt und gesondert hervorgehoben (Supplement 4).

Für die Auswertung wurde die Software MAXQDA 2020 (MAXQDA 2020 Analytics Pro Copyright © 1995–2021, VERBI GmbH) genutzt. Es wurden die Art des Einflusses (Erfolgsfaktoren, Hindernisse, indifferente Einflussfaktoren), die Verantwortlichkeit (Studierendenseite, Dozierendenseite, Manual, Kompetenzorientierung, Organisation, Sonstige) sowie Einzelfaktoren (konkrete Lernziele/Kompetenzen, Vorerfahrung, Engagement, Zeit, Betreuung, Leistungskontrolle u.v.m.) codiert. Nach einer Sättigung der Antworten (weitere Interviews führen voraussichtlich zu keinen neuen Erkenntnissen) wurden keine weiteren Interviews mehr geführt. Die Aussagen aus den Kategorien wurden paraphrasiert und in Kernaussagen zusammengefasst. Sie wurden mit exemplarischen Aussagen belegt und mit Nennung von Häufigkeiten quantifiziert [15]. Die Daten der Studierenden und Dozierenden wurden getrennt voneinander ausgewertet.

## Ergebnisse

### Fragebogenerhebung

**Abb. 1:**
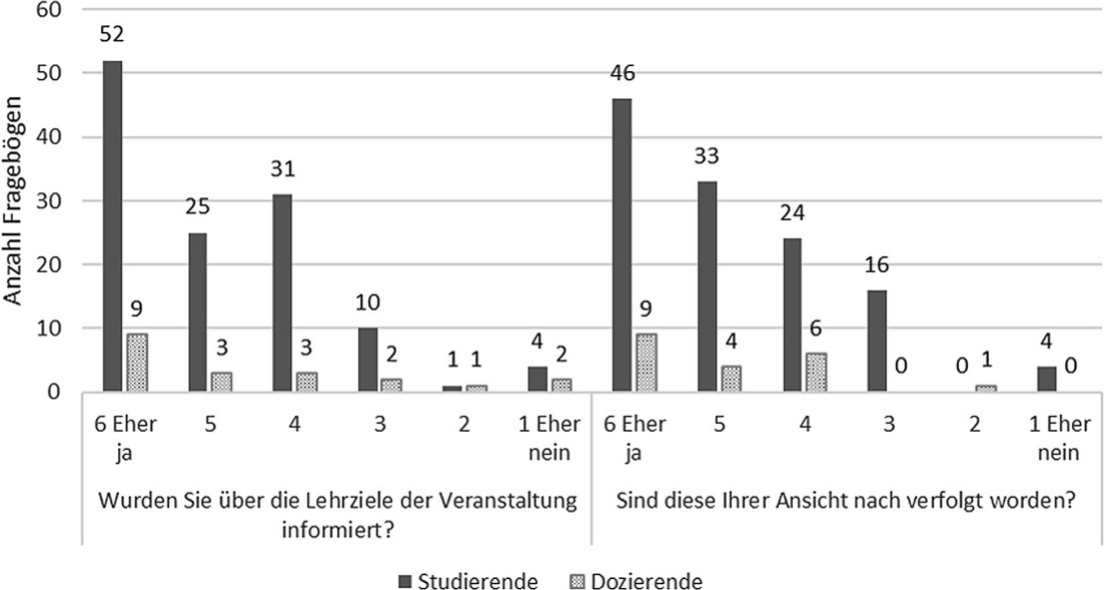
Antworten der Studierenden/Dozierenden zur Information/Verfolgung der Lernziele

Insgesamt 123 Studierendenfragebögen und alle 20 Dozierendenfragebögen lagen vollständig zur Auswertung vor. Die Fragebogenerhebung ergab, dass Studierende und Dozierende mehrheitlich über die Lernziele informiert waren (Likert-Skala 6–4; 88 % bzw. 75 %) und diese als verfolgt ansahen (84 % bzw. 95 %; Abb. 1).

**Abb. 2:**
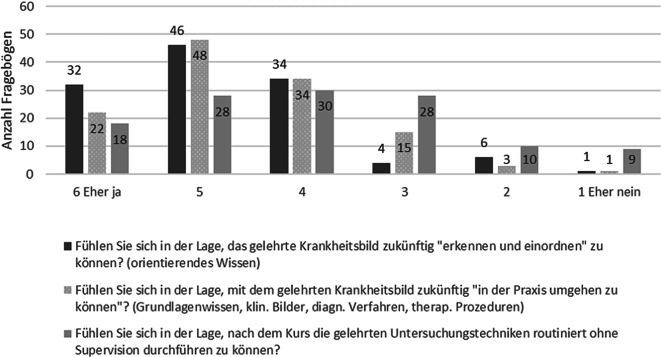
Antworten der Studierenden zum Kompetenzerwerb auf unterschiedlichen Kompetenzebenen

Bei der expliziten Nachfrage, ob Kompetenzen in unterschiedlichen Bereichen vermittelt wurden, wurde dieses mehrheitlich eher bejaht als verneint. In Bezug auf die Anamneseerhebung bejahten (Likert-Skala 6–4) dies 85 %, die klinische Untersuchung 90 % und die Entwicklung eines Behandlungsplans 90 % der Dozierenden. Bei den Studierenden bejahten (Likert-Skala 6–4) dies für die Anamneseerhebung 87 %, für die klinische Untersuchung 89 % und für die Entwicklung eines Behandlungsplans 83 %. Ebenso wurde ein subjektiv eingeschätzter Kompetenzerwerb bejaht, wobei sie eher die Kompetenzebenen 1 (91 %) und 2 (85 %) und weniger die Kompetenzebene 3 (62 %) erreicht haben (Abb. 2). Auch die Dozierenden gaben an, dass nach ihrer Einschätzung ein Kompetenzerwerb auf Seiten der Studierenden stattgefunden hat. Für die Ebene 1 wurde dies mit 100 % bejaht (Likert-Skala 4–6), auf Ebene 2 mit 95 % und auf Ebene 3 mit 75 %.

### Fokusgruppeninterviews

**Abb. 3:**
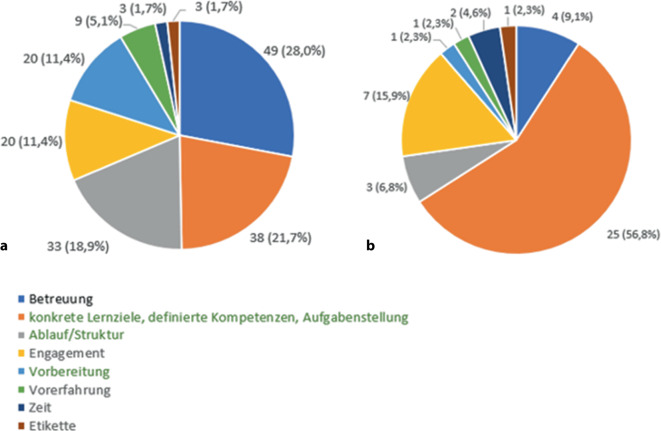
Übersicht Erfolgsfaktoren (**a** Studierenden- und **b** Dozierendeninterviews). (Faktoren in Abhängigkeit von Kompetenzorientierung sind *grün* dargestellt)

Insgesamt nahmen 31 Studierende und 14 Lehrende mit unterschiedlichen Vorerfahrungen (Studierende in der Chirurgie und Lehrende mit Lehrerfahrung) an den Fokusgruppeninterviews teil. Die Studierenden nannten als Erfolgsfaktoren die Betreuung (28 %), konkrete Lernziele/definierte Kompetenzen (22 %) und Ablauf/Struktur des Unterrichts (19 %). Die Dozierenden gaben ebenfalls das Vorhandensein konkreter Lernziele/Kompetenzen (57 %) sowie das Engagement der Studierenden (16 %) als zentrale Elemente des Erfolges an (Abb. 3).

**Abb. 4:**
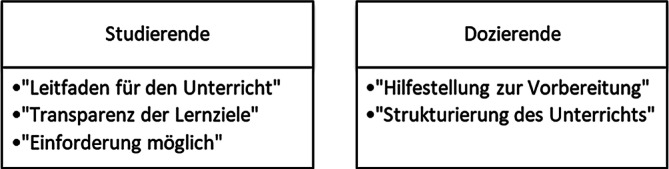
Zentral genannte Aspekte der Erfolgsfaktoren

Während des Interviews kristallisierten sich zentrale Aspekte auf Seiten der Studierenden und Dozierenden als Erfolgsfaktoren für die Implementierung kompetenzorientierter Lehre heraus (Abb. 4). Seitens der Studierenden lautete eine Aussage diesbezüglich:

„Also mir hats insofern geholfen, man wusste zumindest was so die Lernziele sind, das hat auf jeden Fall was gebracht und man hatte so einen kleinen Leitfaden für sich, was man vielleicht noch nicht gemacht hat und wo man vielleicht noch mal gucken muss, dass man es irgendwie wahrnimmt.“ (FG1, Pos. 32)

**Abb. 5:**
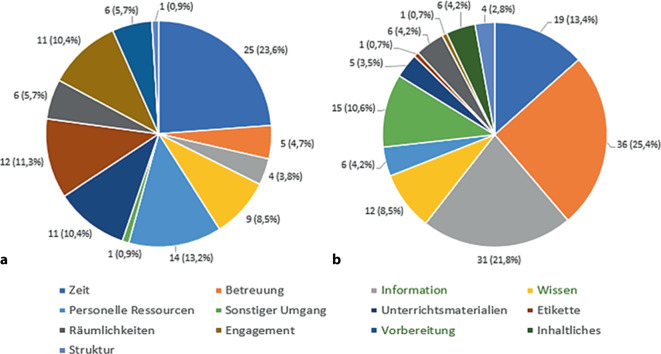
Übersicht der Hindernisse (**a** Studierenden- und **b** Dozierendeninterviews) im interfakultären Vergleich. (Faktoren in Abhängigkeit von Kompetenzorientierung sind *grün* dargestellt)

Als Hindernisse ließen sich insbesondere die „Betreuung“ durch die Dozierenden als auch die „Zeit“ für die Studierenden herausarbeiten (Abb. 5). In „Abhängigkeit von Kompetenzorientierung“ beklagten die Studierenden Aspekte der fehlenden Information oder eines „Informationschaos“. Die Dozierenden nannten insbesondere die „fehlende Zeit für die Lehre“, den „parallelen Klinikalltag“, welche jedoch unabhängig der Kompetenzorientierung wirkten. Hindernisse, die in Abhängigkeit der Kompetenzorientierung wirkten, wurden z. B. seitens der Dozierenden geäußert:

„(…), problematisch war dann aber teilweise, dass bedingt durch den Lernzielkatalog eine Fülle an Lernzielen da waren, ähm so dass die Studierenden dann meist in diesen Einheiten, in denen sie da sind, Ziele gar nicht erreichen konnten.“ (FG11, Pos. 13)

Als indifferente Einflussfaktoren (Tab. 1) wurden verschiedene Begriffe, die in Abhängigkeit der stattgehabten Veränderung wirkten, unter den Kategorien „Manual“, „Leistungs-(Lernziel‑)Kontrolle“, „Kompetenzorientierung“ und „Kompetenztiefe“ zusammengefasst (s. auch Supplement 4).

**Tab. 1:** Übersicht über indifferente Einflussfaktoren (Studierenden- und Dozierendeninterviews)

Indifferente Einflussfaktoren	Dozierendeninterviews	Studierendeninterviews
	*n* (%)	*n* (%)
*Manual*	3 (15,0)	12 (35,5)
*Leistungs-(Lernziel‑)Kontrolle*	4 (20,0)	5 (14,7)
*Kompetenzorientierung*	6 (30,0)	13 (38,2)
*Kompetenztiefe*	3 (15,0)	–

Faktoren in Abhängigkeit von Kompetenzorientierung sind *kursiv* dargestellt

Die Studierenden sprachen den Dozierenden die Hauptverantwortlichkeit (40 %) für den Erfolg kompetenzorientierter Lehre zu wie auch dem begleitenden kompetenzorientierten Manual (20 %). Dozierende wiederum sprachen dem kompetenzorientierten Manual die Hauptverantwortlichkeit Verantwortlichkeit (38 %) zu. Die „Organisation“ trug mit ähnlicher Gewichtung in beiden Gruppen eine Hauptverantwortlichkeit für das Nichtgelingen.

## Diskussion

Die vorliegende Arbeit beschreibt erstmals, nach stattgehabter kompetenzorientierter Veränderung in den klinischen Kursen „Unterricht am Krankenbett Chirurgie“ und „Blockpraktikum Chirurgie“ an zwei Fakultäten, die Erfolgsfaktoren und Hindernisse bei der Implementierung kompetenzorientierter Lehre aus Sicht der Studierenden und Dozierenden. Hierbei sind diese teilweise als abhängig von der stattgehabten Veränderung und teilweise als immanent und unabhängig von dieser zu werten.

Die Auswertung der Fokusgruppeninterviews konnte aufzeigen, dass durch die kompetenzorientierten Veränderungen eine Zunahme an Transparenz der Lehr‑/Lernziele für die Studierenden und Dozierenden gelingt, welche als positiv erlebt wird. Auflistung und Ausarbeitung in begleitenden Manualen/Portfolios stellen hier eine Verbesserung dar und ermöglichen beiden Seiten eine Fokussierung auf das Erreichen der Lernziele und Übertragen zugleich die Verantwortlichkeit dafür auf Seiten der Studierenden und der Dozierenden. Ähnlich beschrieben König et al., dass, durch das Vorhandensein gleichermaßen leicht begreif- und verfügbarer Lehr‑/Lernzielen für Dozierende und Studierende, letztlich der Lernprozess unabhängig der Lehrperson in den Vordergrund rücken kann [12]. Dies erscheint essenziell in Anbetracht der fehlenden Kontinuität durch feste Lehrpersonen im klinischen Alltag. Die Dozierenden berichten auch im Rahmen unserer Fokusgruppeninterviews, dass ihnen das Manual und die konkreten Lernziele eine Hilfestellung sind, wenn sie spontan den Unterricht übernehmen müssen und sie daran den Unterricht strukturieren können. Diese Ergebnisse sind als Erfolg der kompetenzorientierten Veränderung zu werten und haben den Nebeneffekt, dass selbstregulierendes Lernen gefördert wird, welches wiederum einen zentralen Grundgedanken der kompetenzorientierten Lehre darstellt [25], und somit durch die stattgehabte Veränderung verfolgt werden konnte. Eine konstante Betreuung (Mentor), Engagement auf Seite der Dozierenden und aktives Einbinden der Studierenden stellen ferner Erfolgsfaktoren für die Implementierung kompetenzorientierter Lehre dar, welche jedoch als unabhängig der kompetenzorientierten Veränderung zu werten sind. Umgekehrt geben die Dozierenden die aktive Teilnahme und das Interesse auf Seiten der Studierenden maßgeblich als Erfolgsfaktor an. Diesen positiven Einfluss konnten Dybowski et al. bereits 2017 im Rahmen einer Untersuchung der Lehrqualität beeinflussenden Faktoren nachweisen [5]. Rüsseler et al. beschrieben, dass Studierende und Lehrende angaben, dass durch ein Fehlen definierter Ausbildungsziele die Lehre stark von der Motivation und den Interessen der Beteiligten abhinge und dass Studierende ihren Kompetenzstand nicht einschätzen könnten [18]. Die Ergebnisse sowohl der Fragebogenerhebung als auch der Fokusgruppeninterviews scheinen, durch die Implementierung der kompetenzorientierten Lehre, für eine Verbesserung dieser Aspekte zu sprechen. Insbesondere im Rahmen der Fragebogenerhebung wurde ein stattgefundener Kompetenzerwerb durch die Studierenden angegeben, welcher abhängig von der Kompetenzebene war. Je niedriger diese war, desto mehr Studierende gaben an, die entsprechende Kompetenz erworben zu haben. Dozierende gaben Zeit- und Personalmangel als maßgebliche Hindernisse an. Ähnliches wurde auch von Sterz et al. beschrieben, welche im Rahmen einer Interviewerhebung Dozierende u. a. zu Hemmnissen für die Lehre im klinischen Alltag befragten. Im Rahmen dieser Befragung wurde zudem auch die geringe Motivation der Studierenden als Hemmnis identifiziert [24]. Vergleichbare Aussagen „Studierende tauchen irgendwann auf“, „stellen sich nicht vor“ wurden auch bei den Interviews der Dozierenden in der vorliegenden Arbeit als Hindernisse genannt. Dozierende äußerten im Rahmen dieser Befragung den Wunsch, die Lehre unabhängig des Klinikalltags durchführen zu können. Gleiches gaben auch die Studierenden an und wünschen sich Dozierenden, deren Telefon ausgeschaltet ist und die nicht nebenbei Patienten versorgen müssen. Schrauth et al. beschrieben diese Erwartungshaltung seitens der Studierenden im Rahmen einer Analyse mit Studierenden im Praktischen Jahr bereits 2009 [22]. Die Freistellung von anderen Tätigkeiten wurde auch von Kiefer et al. in einer Befragung von Dozierenden zu möglichen Verbesserungen der universitären Lehre, als gewünschter Anreiz für gute Lehre, neben besserer Entlohnung und höherer Mittelzuweisung, genannt [11]. Studierende beschreiben die schlechte Organisation der Kliniken, fehlende freie Zeiten für die Lehrtätigkeit und Geringschätzung der Lehre als solche als Ursachen für die fehlende Motivation von Dozierenden [18]. Auch wenn ein Mangel an didaktischer Ausbildung insbesondere bei AssistenzärztInnen beklagt wird [13] und didaktische Trainings und Techniken essenziell sind [1], stellte dies im Rahmen unserer Untersuchung keinen beeinflussenden Faktor dar. Die genannten Hindernisse sind jedoch unabhängig der kompetenzorientierten Veränderung, sondern vielmehr als vom Lehrformat unabhängiges Problem zu werten und scheinen sich auch durch die kompetenzorientierten Veränderungen nicht zu verbessern. Die Dozierenden berichteten, dass durch die transparente Darstellung der Lernziele und zu erreichenden Kompetenzen eine Hilfestellung bestünde, wenn Unterricht spontan übernommen werden muss. Dies ist als Verbesserung durch die kompetenzorientierte Veränderung zu werten, stellt jedoch keine allumfassende Problemlösung dar.

Während des Codierungsprozesses wurde eine Vielzahl von Faktoren als „indifferent“ codiert. Dies entstand daraus, dass keine eindeutige Zuordnung zum Positiven wie Negativen anhand der getätigten Aussage im Kontext möglich war und diese häufig wertfrei geäußert wurde. Gerade die als „indifferent“ codierten Segmente bezogen sich jedoch häufig auf kompetenzabhängige Veränderungen. Darunter sind viele Aspekte, welche die Implementierung kompetenzorientierter Lehre negativ beeinflussen. Eine „Unkenntnis des Manuals“ z. B. ist als Hindernis zu betrachten, wenn die Kompetenzorientierung mithilfe dessen implementiert werden soll. Hierin liegt eine organisatorische Verantwortlichkeit der Fakultäten (lehrenden Kliniken). Wird es nicht genutzt, z. B. weil der Dozierende es nicht nutzen möchte, ist dies ebenfalls der Implementierung nicht förderlich und Schulungen und Aufklärungen der Dozierenden sollten dafür Sorge tragen, dass eine Anwendung stattfindet. Wird es von den Studierenden nicht genutzt, ist auch hier Erörterung des Hintergrundes für die Nutzung sinnvoll. Die „Unsicherheit, was Kompetenzorientierung eigentlich ist“, ist an dieser Stelle am ehesten dem frühen Stadium der Implementierung und dem erstmaligen Kontakt mit dieser geschuldet. Sie zeigt jedoch auf, dass eine Erörterung und intensive Aufklärung für Studierende und Dozierende vonnöten ist, um erfolgreich umgesetzt werden zu können. Auch wenn die Fragebogenerhebung ergab, dass mehrheitlich die Dozierenden der Meinung waren, dass die Studierenden die geforderten Kompetenzen während ihres Kurses erworben hatten, gaben die Dozierenden während der Fokusgruppeninterviews an, dass eine Unsicherheit diesbezüglich, ohne geforderte Abschlussprüfung, bestünde. Diesbezüglich merkte Ahlers in seiner Habilitationsschrift mit Verweis auf entsprechende Quellen jedoch an, dass die Prüfung von Kompetenzen im klinischen Kontext schwer standardisierbar und mit großem Aufwand verbunden sei [3]. Vor diesem Hintergrund bleiben wir die vollständige Umsetzung kompetenzorientierter Lehre inklusive entsprechender Prüfungsleistung schuldig. Diese ist in der zukünftigen Lehrgestaltung und weiterführenden Studien sicher notwendig. Jedoch ging es im Rahmen dieser Untersuchung zunächst um die Implementierung der Kompetenzorientierung in den alltäglichen chirurgischen Lehreinheiten und die Identifikation der Erfolgsfaktoren und Hindernisse dabei.

## Limitationen

Als limitierender Faktor für die Aussagekraft der vorliegenden Arbeit ist zum einen die kurze Dauer der Implementierung der kompetenzorientierten Lehre zu nennen. Es ist anzunehmen, dass durch einen längeren Anwendungszeitraum einige Einflussfaktoren (u. a. Informationsfluss, Unsicherheit bez. der Kompetenzorientierung) eine andere Gewichtung bekommen könnten. Die angewandte statistische Aufarbeitung der Aussagen ist streng genommen nur eingeschränkt zulässig, da hier eine Quantifizierung qualitativer Daten als ergänzender Schritt erfolgt ist. Es fand keine explizite Überprüfung statt, ob nicht getätigte Aussagen aus Gründen des Nichtvorhandenseins oder des Nichterwähnens weggelassen wurden. Ebenfalls ist die durch uns vorgenommene Quantifizierung qualitativer Daten in dessen Methodik eigentlich nicht vorgesehen, sondern durch uns ergänzend mit dem Ziel erfolgt, einen verständlichen Überblick über die Relevanz der einzelnen Aspekte zu geben, aus denen v. a. KlinikerInnen Rückschlüsse für die eigene Umsetzung ziehen können. Die Studie lässt ferner keine valide Aussage über den stattgefundenen Kompetenzerwerb zu, hierfür bedarf es einer entsprechenden Prüfungssituation nach Durchführung der Lehre im Studiendesign. Auch wenn die Ergebnisse nahelegen, dass die Implementierungshindernisse und -erfolgsfaktoren unabhängig von der Art der kompetenzorientierten Veränderungen der beiden eingeschlossenen Fakultäten waren, ist die Aussage über ihre Allgemeingültigkeit nur eingeschränkt möglich, da die Implementierungswege sich an beiden Fakultäten leicht unterschieden. Als weitere Limitation ist anzuführen, dass die qualitative Inhaltsanalyse immer auch einer subjektiven Codierung unterliegt. Eine Codierung desselben Textes durch mehrere Personen erfolgte im Rahmen dieser Untersuchung nicht, sodass dies theoretisch zu veränderten Ergebnissen führen könnte.

## Fazit für die Praxis

Zusammenfassend zeigen die Ergebnisse der vorliegenden Untersuchung auf, dass die klare Strukturierung, die transparenten Lernziele und die funktionierende Betreuung als Erfolgsfaktoren für die Implementierung zu nennen sind. Die Menge der Lernziele stellt hingegen ein Hindernis dar, was bedeutet, dass Lernziele bewusst für Kursformate/-zeit ausgewählt werden sollten. Durch Schulung und Information zum Lernprozess sollten indifferente Faktoren wie „Unklarheit der Kompetenzorientierung“, „Unsicherheit ob Kompetenzerwerb gelungen“ adressiert werden. Die sichtbare Kompetenzorientierung bietet den Dozierenden den Rahmen für die Vermittlung der Lernziele und den Studierenden wird das konkrete Einfordern der Lernziele ermöglicht. Die wesentlichen Hindernisse liegen fern der Kompetenzorientierung bei Zeit, Personalmangel und bei unorganisiertem Informationsfluss. Letzteres ist durch eine einheitliche Kommunikation zu verbessern. Die immanenten Probleme des Zeit- und Personalmangels beeinflussen leider auch die Implementierung der Kompetenzorientierung und bedürfen einer generellen strukturellen Veränderung.

## Supplementary Information

Supplement 1: Fragebögen Studierende/Dozierende

Supplement 2: Interviewleitfäden Dozierende/Studierende

Supplement 3: Übersicht über deduktiv und induktiv gebildete Kategorien

Supplement 4: Codierungsmöglichkeiten mit dem Schwerpunkt auf die Kompetenzorientierung
