# Supplementary material for: Success factors and obstacles in the implementation of competence-oriented teaching in surgery
Source: Chirurgie (Heidelb). 2024 Jun 3;95(10):833–40. [Article in German] doi: 10.1007/s00104-024-02107-9 (PMC11413039; doi:10.1007/s00104-024-02107-9)
Supplement: Supplementary file 1 — Supplement 1: Fragebögen Studierende/Dozierende [file 104_2024_2107_MOESM1_ESM.docx]

**Supplement 1: Fragebögen Studierende /Dozierende**

Studierende (Jeweils Anpassung des Textes an UAK, Blockpraktikum)

|  | **Likert-skala**  **(6=eher ja, 1= eher nein)** |
| --- | --- |
| **Frage** | **Eher ja – eher nein** |
| **Allgemein** |  |
| War der Dozierende auf die Veranstaltung vorbereitet? | 6 – 5 - 4 – 3 – 2 – 1 |
| War der Unterricht klar strukturiert? | 6 – 5 - 4 – 3 – 2 - 1 |
| Wurden Sie über die Lehrziele der Veranstaltung informiert? | 6 – 5 - 4 – 3 – 2 - 1 |
| Wurden diese Ihrer Ansicht nach verfolgt? | 6 – 5 - 4 – 3 – 2 – 1 |
| Haben Sie das begleitende Manual für Ihre Vor-oder Nachbereitung genutzt? | 6 – 5 - 4 – 3 – 2 – 1 |
| Sind Ihnen Kompetenzen vermittelt worden in den Bereichen:  - Anamneseerhebung  - klinische Untersuchung  - Entwicklung eines Behandlungsplans | 6 – 5 - 4 – 3 – 2 – 1  6 – 5 – 4 – 3 – 2 – 1  6 – 5 – 4 – 3 – 2 - 1 |
| **Theoretisches Wissen** |  |
| Fühlen Sie sich in der Lage das gelehrte Krankheitsbild zukünftig „erkennen und einordnen“ zu können? (orientierendes Wissen) | 6 – 5 - 4 – 3 – 2 – 1 |
| Fühlen Sie sich in der Lage mit dem gelehrten Krankheitsbild zukünftig „in der Praxis umgehen zu können“? (Grundlagenwissen, klin. Bilder, diagn. Verfahren, therap. Prozeduren) | 6 – 5 - 4 – 3 – 2 – 1 |
| **Skills** |  |
| Haben Sie theoretisches Wissen bzgl. Untersuchungstechniken zugehörig zum gelehrten Krankheitsbild vermittelt bekommen? | 6 – 5 - 4 – 3 – 2 – 1 |
| Haben Sie die Untersuchungstechniken zugehörig zum gelehrten Krankheitsbild gesehen und demonstriert bekommen? | 6 – 5 - 4 – 3 – 2 – 1 |
| Haben Sie die Untersuchungstechniken zugehörig zum gelehrten Krankheitsbild selbstständig angewendet und durchgeführt? | 6 – 5 - 4 – 3 – 2 – 1 |
| Fühlen Sie sich in der Lage nach dem Kurs die gelehrten Untersuchungstechniken routiniert ohne Supervision durchführen zu können? | 6 – 5 - 4 – 3 – 2 - 1 |

Bitte die Antwort mit einem **X** oder einem **O** markieren.

Dozierende (Jeweils Anpassung des Textes an UAK, Blockpraktikum)

|  | **Likert-skala**  **(6=eher ja, 1= eher nein)** |
| --- | --- |
| **Allgemein** |  |
| Wie häufig haben Sie während des Sommersemesters den Unterricht am Krankenbett (UAK) unterrichtet? | 1-3 3-6 6-10 >10 Kurse |
|  | **Eher ja - eher nein** |
| Hatten Sie mehrheitlich ausreichend Zeit sich auf den Kurs vorzubereiten? | 6 – 5 - 4 – 3 – 2 - 1 |
| Wurden Sie über die Lehrziele der Veranstaltung informiert? | 6 – 5 - 4 – 3 – 2 - 1 |
| Sind diese Ihrer Ansicht nach verfolgt worden? | 6 – 5 - 4 – 3 – 2 – 1 |
| Haben Sie schon häufig das Format UAK unterrichtet? | 6 – 5 - 4 – 3 – 2 – 1 |
| Haben Sie das begleitende Manual für Ihre Vor-oder Nachbereitung genutzt? | 6 – 5 - 4 – 3 – 2 – 1 |
| Glauben Sie, dass Sie Kompetenzen vermitteln konnten in den Bereichen:  - Anamneseerhebung  - klinische Untersuchung  - Entwicklung eines Behandlungsplans | 6 – 5 - 4 – 3 – 2 – 1  6 – 5 – 4 – 3 – 2 – 1  6 – 5 – 4 – 3 – 2 - 1 |
| **Theoretisches Wissen** |  |
| Haben Sie den Eindruck, dass die Studierenden nach ihrem Kurs in der Lage sind das gelehrte Krankheitsbild zukünftig „erkennen und einordnen“ zu können? (orientierendes Wissen) | 6 – 5 - 4 – 3 – 2 – 1 |
| Haben Sie den Eindruck, dass die Studierenden nach ihrem Kurs in der Lage mit dem gelehrten Krankheitsbild zukünftig „in der Praxis umgehen zu können“? (Grundlagenwissen, klin. Bilder, diagn. Verfahren, therap. Prozeduren) | 6 – 5 - 4 – 3 – 2 – 1 |
| **Skills** |  |
| Waren Sie in der Lage theoretisches Wissen bzgl. Untersuchungstechniken zugehörig zum gelehrten Krankheitsbild den Studierenden vermitteln zu können? | 6 – 5 - 4 – 3 – 2 – 1 |
| Haben Sie im Rahmen ihres UAKs die Untersuchungstechniken zugehörig zum gelehrten Krankheitsbild den Studierenden demonstriert? | 6 – 5 - 4 – 3 – 2 – 1 |
| Haben die Studierenden im Rahmen ihres UAKs die Untersuchungstechniken zugehörig zum gelehrten Krankheitsbild selbstständig angewendet und durchgeführt? | 6 – 5 - 4 – 3 – 2 – 1 |
| Sind die Studierenden, Ihrer Einschätzung nach, nach Ihrem UAK die gelehrten Untersuchungstechniken routiniert ohne Supervision durchführen zu können? | 6 – 5 - 4 – 3 – 2 - 1 |

Bitte die Antwort mit einem **X** oder einem **O** markieren.
