# Supplementary material for: Success factors and obstacles in the implementation of competence-oriented teaching in surgery
Source: Chirurgie (Heidelb). 2024 Jun 3;95(10):833–40. [Article in German] doi: 10.1007/s00104-024-02107-9 (PMC11413039; doi:10.1007/s00104-024-02107-9)
Supplement: Supplementary file 2 — Supplement 2: Interviewleitfäden Dozierende/Studierende [file 104_2024_2107_MOESM2_ESM.docx]

**Supplement 2: Interviewleitfäden Dozierende / Studierende**

Interviewleitfäden UAK Bochum (Studierenden Interview I)

**Allgemein** (Geschlecht, Alter, Berufserfahrung im Gesundheitswesen)

1. Sind die bisher absolvierten Termine des Kurses „UAK Chirurgie“ aus Ihrer Sicht erfolgreich verlaufen?
   1. Wenn ja, warum?
   2. Wenn nein, warum nicht?

**Studentische Seite**

1. Welche Faktoren **auf Studierendenseite** haben sich negativ ausgewirkt, welche positiv?

*(z.B. Vorbereitung, Recherche, Manual, Moodle, Gruppendynamik, Kompetenztiefe…)*

**Seite der Dozierenden**

1. Welche Faktoren **auf der Seite der Dozierenden** haben sich positiv ausgewirkt, welche negativ?

*(z.B. Pünktlichkeit, Vorbereitung, Organisation, Freundlichkeit, Fachliche/didaktisch.)*

**Umstände**

1. Gab es äußere Umstände, die den Erfolg des Kurses maßgeblich negativ oder positiv beeinflusst haben *(z.B. Patientenkontakt, Räumlichkeiten, medizinisches Personal, Zeit, Unterrichtsmaterialien etc.)*
   1. Wenn ja, welche waren das?

**Begleitendes kompetenzorientiertes Manual zum UAK**

1. Haben Sie das kompetenzorientierte Manual zum UAK angewendet?
   1. Wenn nein, warum nicht?
2. Wenn ja, hatte das kompetenzorientierte Manual zum UAK einen Einfluss auf den Ablauf des Kurses im positiven oder negativen Sinne? *(z.B. Strukturierung, Transparenz, klare Inhalte/Ziele) (z.B. Leistungsdruck, Reduktion auf Kompetenzen)*
   1. Wenn ja, warum bzw. welchen?
   2. Wenn nein, warum nicht?
3. Hat das kompetenzorientierte Manual bei einem spezifischen Thema/Situation geholfen? *(z.B. Unterrichtssituation, Thema, Tätigkeit, …)*
4. Ist es Ihnen bewusst welches Ziel mit der Kompetenzorientierung des Unterrichts verfolgt wird.
   1. Bitte erläutern Sie ihre Antwort
5. War die vorgegebene Kompetenztiefe Ihrer Meinung nach angemessen und konnten Sie diese erreichen?
   1. Wenn nein, warum nicht?
6. Hatte die Kompetenzorientierung im Vergleich zu anderen Kursen ohne diese Ausrichtung einen Vorteil oder Nachteil?
   1. Bitte erläutern Sie ihre Antwort
7. Haben Sie das Gefühl, dass sie das Lernziel erreicht haben?
   1. Bitte erläutern Sie ihre Antwort

**Abschluss**

1. Was wäre ihrer Ansicht nach notwendig, um den Kurs für Sie zu verbessern?

**Dozierenden Interview II**

**Allgemein** (Geschlecht, Alter, Ass/FA/OA/Didaktische Vorerfahrung (Medizindidaktik etc.))

1. Sind die bisher absolvierten Termine des Kurses „UAK Chirurgie“ aus Ihrer Sicht erfolgreich verlaufen?
   1. Wenn ja, warum?
   2. Wenn nein, warum nicht?

**Studentische Seite**

1. Welche Faktoren **auf Studierendenseite** haben sich negativ ausgewirkt, welche positiv?

*(z.B. Vorbereitung, Recherche, Manual, Moodle, Gruppendynamik, Kompetenztiefe…)*

**Seite der Dozierenden**

1. Welche Faktoren **auf der Seite der Dozierenden** haben sich positiv ausgewirkt, welche negativ?

*(z.B. Pünktlichkeit, Vorbereitung, Organisation, Freundlichkeit, Fachliche/didaktisch…)*

**Umstände**

1. Gab es äußere Umstände, die den Erfolg des Kurses maßgeblich negativ oder positiv beeinflusst haben *(z.B. Patientenkontakt, Räumlichkeiten, medizinisches Personal, Zeit, Unterrichtsmaterialien etc.)*
   1. Wenn ja, welche waren das?

**Begleitendes kompetenzorientiertes Manual zum UAK**

1. Haben Sie das kompetenzorientierte Manual zum UAK angewendet?
   1. Wenn nein, warum nicht?
2. Wenn ja, hatte das kompetenzorientierte Manual zum UAK einen Einfluss auf den Ablauf des Kurses im positiven oder negativen Sinne? *(z.B. Strukturierung, Transparenz, klare Inhalte/Ziele) (z.B. Leistungsdruck, Reduktion auf Kompetenzen)*
   1. Wenn ja, warum bzw. welchen?
   2. Wenn nein, warum nicht?
3. Hat das kompetenzorientierte Manual bei einem spezifischen Thema/Situation geholfen? *(z.B. Unterrichtssituation, Thema, Tätigkeit, …)*
4. Ist es Ihnen bewusst welches Ziel mit der Kompetenzorientierung des Unterrichts verfolgt wird.
   1. Bitte erläutern Sie ihre Antwort
5. War die vorgegebene Kompetenztiefe Ihrer Meinung nach angemessen und konnten die Studierenden diese erreichen?
   1. Wenn nein, warum nicht?
6. Hatte die Kompetenzorientierung im Vergleich zu anderen Kursen ohne diese Ausrichtung einen Vorteil oder Nachteil?
   1. Bitte erläutern Sie ihre Antwort
7. Haben Sie das Gefühl, dass sie das die Studierenden das Lernziel erreicht haben?
   1. Bitte erläutern Sie ihre Antwort

**Abschluss**

1. Was wäre ihrer Ansicht nach notwendig, um den Kurs für Sie zu verbessern?

Interviewleitfäden Blockpraktikum Bochum (Studierenden Interview I)

**Allgemein** (Geschlecht, Alter, Berufserfahrung im Gesundheitswesen)

1. Sind die bisher absolvierten Termine des Kurses „Blockpraktikum Chirurgie“ aus Ihrer Sicht erfolgreich verlaufen?
   1. Wenn ja, warum?
   2. Wenn nein, warum nicht?

**Studentische Seite**

1. Welche Faktoren **auf Studierendenseite** haben sich negativ ausgewirkt, welche positiv?

*(z.B. Vorbereitung, Recherche, Manual, Moodle, Gruppendynamik, Kompetenztiefe…)*

**Seite der Dozierenden**

1. Welche Faktoren **auf der Seite der Dozierenden** haben sich positiv ausgewirkt, welche negativ?

*(z.B. Pünktlichkeit, Vorbereitung, Organisation, Freundlichkeit, Fachliche/didaktisch..)*

**Umstände**

1. Gab es äußere Umstände, die den Erfolg des Kurses maßgeblich negativ oder positiv beeinflusst haben *(z.B. Patientenkontakt, Räumlichkeiten, medizinisches Personal, Zeit, Unterrichtsmaterialien etc.)*
   1. Wenn ja, welche waren das?

**Begleitendes kompetenzorientiertes Manual zum Blockpraktikum**

1. Haben Sie das kompetenzorientierte Manual zum Blockpraktikum angewendet?
   1. Wenn nein, warum nicht?
2. Wenn ja, hatte das kompetenzorientierte Manual zum Blockpraktikum einen Einfluss auf den Ablauf des Kurses im positiven oder negativen Sinne? *(z.B. Strukturierung, Transparenz, klare Inhalte/Ziele) (z.B. Leistungsdruck, Reduktion auf Kompetenzen)*
   1. Wenn ja, warum bzw. welchen?
   2. Wenn nein, warum nicht?
3. Hat das kompetenzorientierte Manual bei einem spezifischen Thema/Situation geholfen? *(z.B. Unterrichtssituation, Thema, Tätigkeit, …)*
4. Ist es Ihnen bewusst welches Ziel mit der Kompetenzorientierung des Unterrichts verfolgt wird.
   1. Bitte erläutern Sie ihre Antwort
5. War die vorgegebene Kompetenztiefe Ihrer Meinung nach angemessen und konnten Sie diese erreichen?
   1. Wenn nein, warum nicht?
6. Hatte die Kompetenzorientierung im Vergleich zu anderen Kursen ohne diese Ausrichtung einen Vorteil oder Nachteil?
   1. Bitte erläutern Sie ihre Antwort
7. Haben Sie das Gefühl, dass sie das Lernziel erreicht haben?
   1. Bitte erläutern Sie ihre Antwort

**Abschluss**

1. Was wäre ihrer Ansicht nach notwendig, um den Kurs für Sie zu verbessern?

**Dozierenden Interview II**

**Allgemein** (Geschlecht, Alter, Ass/FA/OA/Didaktische Vorerfahrung (Medizindidaktik etc.))

1. Sind die bisher absolvierten Termine des Kurses „Blockpraktikum Chirurgie“ aus Ihrer Sicht erfolgreich verlaufen?
   1. Wenn ja, warum?
   2. Wenn nein, warum nicht?

**Studentische Seite**

1. Welche Faktoren **auf Studierendenseite** haben sich negativ ausgewirkt, welche positiv?

*(z.B. Vorbereitung, Recherche, Manual, Moodle, Gruppendynamik, Kompetenztiefe…)*

**Seite der Dozierenden**

1. Welche Faktoren **auf der Seite der Dozierenden** haben sich positiv ausgewirkt, welche negativ?

*(z.B. Pünktlichkeit, Vorbereitung, Organisation, Freundlichkeit, Fachliche/didaktisch..)*

**Umstände**

1. Gab es äußere Umstände, die den Erfolg des Kurses maßgeblich negativ oder positiv beeinflusst haben *(z.B. Patientenkontakt, Räumlichkeiten, medizinisches Personal, Zeit, Unterrichtsmaterialien etc.)*
   1. Wenn ja, welche waren das?

**Begleitendes kompetenzorientiertes Manual zum Blockpraktikum**

1. Haben Sie das kompetenzorientierte Manual zum Blockpraktikum angewendet?
   1. Wenn nein, warum nicht?
2. Wenn ja, hatte das kompetenzorientierte Manual zum Blockpraktikum einen Einfluss auf den Ablauf des Kurses im positiven oder negativen Sinne? *(z.B. Strukturierung, Transparenz, klare Inhalte/Ziele) (z.B. Leistungsdruck, Reduktion auf Kompetenzen)*
   1. Wenn ja, warum bzw. welchen?
   2. Wenn nein, warum nicht?
3. Hat das kompetenzorientierte Manual bei einem spezifischen Thema/Situation geholfen? *(z.B. Unterrichtssituation, Thema, Tätigkeit, …)*
4. Ist es Ihnen bewusst welches Ziel mit der Kompetenzorientierung des Unterrichts verfolgt wird.
   1. Bitte erläutern Sie ihre Antwort
5. War die vorgegebene Kompetenztiefe Ihrer Meinung nach angemessen und konnten die Studierenden diese erreichen?
   1. Wenn nein, warum nicht?
6. Hatte die Kompetenzorientierung im Vergleich zu anderen Kursen ohne diese Ausrichtung einen Vorteil oder Nachteil?
   1. Bitte erläutern Sie ihre Antwort
7. Haben Sie das Gefühl, dass sie das die Studierenden das Lernziel erreicht haben?
   1. Bitte erläutern Sie ihre Antwort

**Abschluss**

1. Was wäre ihrer Ansicht nach notwendig, um den Kurs für Sie zu verbessern?

Interviewleitfäden Blockpraktikum Essen (Studierenden Interview I)

**Allgemein** (Geschlecht, Alter, Berufserfahrung im Gesundheitswesen)

1. Sind die bisher absolvierten Termine des Kurses „Blockpraktikum Chirurgie“ aus Ihrer Sicht erfolgreich verlaufen?
   1. Wenn ja, warum?
   2. Wenn nein, warum nicht?

**Studentische Seite**

1. Welche Faktoren **auf Studierendenseite** haben sich negativ ausgewirkt, welche positiv?

*(z.B. Vorbereitung, Recherche, Skript, Moodle, Gruppendynamik, Kompetenztiefe…)*

**Seite der Dozierenden**

1. Welche Faktoren **auf der Seite der Dozierenden** haben sich positiv ausgewirkt, welche negativ?

*(z.B. Pünktlichkeit, Vorbereitung, Organisation, Freundlichkeit, Fachliche/didaktisch..)*

**Umstände**

1. Gab es äußere Umstände, die den Erfolg des Kurses maßgeblich negativ oder positiv beeinflusst haben *(z.B. Patientenkontakt, Räumlichkeiten, medizinisches Personal, Zeit, Unterrichtsmaterialien etc.)*
   1. Wenn ja, welche waren das?

**Begleitendes kompetenzorientiertes Portfolio zum Blockpraktikum**

1. Haben Sie das kompetenzorientierte Portfolio zum Blockpraktikum angewendet?
   1. Wenn nein, warum nicht?
2. Wenn ja, hatte das kompetenzorientierte Portfolio zum Blockpraktikum einen Einfluss auf den Ablauf des Kurses im positiven oder negativen Sinne? *(z.B. Strukturierung, Transparenz, klare Inhalte/Ziele) (z.B. Leistungsdruck, Reduktion auf Kompetenzen)*
   1. Wenn ja, warum bzw. welchen?
   2. Wenn nein, warum nicht?
3. Hat das kompetenzorientierte Portfolio bei einem spezifischen Thema/Situation geholfen? *(z.B. Unterrichtssituation, Thema, Tätigkeit, …)*
4. Ist es Ihnen bewusst welches Ziel mit der Kompetenzorientierung des Unterrichts verfolgt wird.
   1. Bitte erläutern Sie ihre Antwort
5. War die vorgegebene Kompetenztiefe Ihrer Meinung nach angemessen und konnten Sie diese erreichen?
   1. Wenn nein, warum nicht?
6. Hatte die Kompetenzorientierung im Vergleich zu anderen Kursen ohne diese Ausrichtung einen Vorteil oder Nachteil?
   1. Bitte erläutern Sie ihre Antwort
7. Haben Sie das Gefühl, dass sie das Lernziel erreicht haben?
   1. Bitte erläutern Sie ihre Antwort

**Abschluss**

1. Was wäre ihrer Ansicht nach notwendig, um den Kurs für Sie zu verbessern?

**Dozierenden Interview II**

**Allgemein** (Geschlecht, Alter, Ass/FA/OA/Didaktische Vorerfahrung (Medizindidaktik etc.))

1. Sind die bisher absolvierten Termine des Kurses „Blockpraktikum Chirurgie“ aus Ihrer Sicht erfolgreich verlaufen?
   1. Wenn ja, warum?
   2. Wenn nein, warum nicht?

**Studentische Seite**

1. Welche Faktoren **auf Studierendenseite** haben sich negativ ausgewirkt, welche positiv?

*(z.B. Vorbereitung, Recherche, Skript, Moodle, Gruppendynamik, Kompetenztiefe…)*

**Seite der Dozierenden**

1. Welche Faktoren **auf der Seite der Dozierenden** haben sich positiv ausgewirkt, welche negativ?

*(z.B. Pünktlichkeit, Vorbereitung, Organisation, Freundlichkeit, Fachliche/didaktisch..)*

**Umstände**

1. Gab es äußere Umstände, die den Erfolg des Kurses maßgeblich negativ oder positiv beeinflusst haben *(z.B. Patientenkontakt, Räumlichkeiten, medizinisches Personal, Zeit, Unterrichtsmaterialien etc.)*
   1. Wenn ja, welche waren das?

**Begleitendes kompetenzorientiertes Portfolio zum Blockpraktikum**

1. Haben Sie das kompetenzorientierte Portfolio zum Blockpraktikum angewendet?
   1. Wenn nein, warum nicht?
2. Wenn ja, hatte das kompetenzorientierte Portfolio zum Blockpraktikum einen Einfluss auf den Ablauf des Kurses im positiven oder negativen Sinne? *(z.B. Strukturierung, Transparenz, klare Inhalte/Ziele) (z.B. Leistungsdruck, Reduktion auf Kompetenzen)*
   1. Wenn ja, warum bzw. welchen?
   2. Wenn nein, warum nicht?
3. Hat das kompetenzorientierte Portfolio bei einem spezifischen Thema/Situation geholfen? *(z.B. Unterrichtssituation, Thema, Tätigkeit, …)*
4. Ist es Ihnen bewusst welches Ziel mit der Kompetenzorientierung des Unterrichts verfolgt wird.
   1. Bitte erläutern Sie ihre Antwort
5. War die vorgegebene Kompetenztiefe Ihrer Meinung nach angemessen und konnten die Studierenden diese erreichen?
   1. Wenn nein, warum nicht?
6. Hatte die Kompetenzorientierung im Vergleich zu anderen Kursen ohne diese Ausrichtung einen Vorteil oder Nachteil?
   1. Bitte erläutern Sie ihre Antwort
7. Haben Sie das Gefühl, dass sie das die Studierenden das Lernziel erreicht haben?
   1. Bitte erläutern Sie ihre Antwort

**Abschluss**

1. Was wäre ihrer Ansicht nach notwendig, um den Kurs für Sie zu verbessern?
