# Supplementary figures and images for: Success factors and obstacles in the implementation of competence-oriented teaching in surgery
Source: Chirurgie (Heidelb). 2024 Jun 3;95(10):833–40. [Article in German] doi: 10.1007/s00104-024-02107-9 (PMC11413039; doi:10.1007/s00104-024-02107-9)

Supplement 3: Übersicht über deduktiv und induktiv gebildeten Kategorien


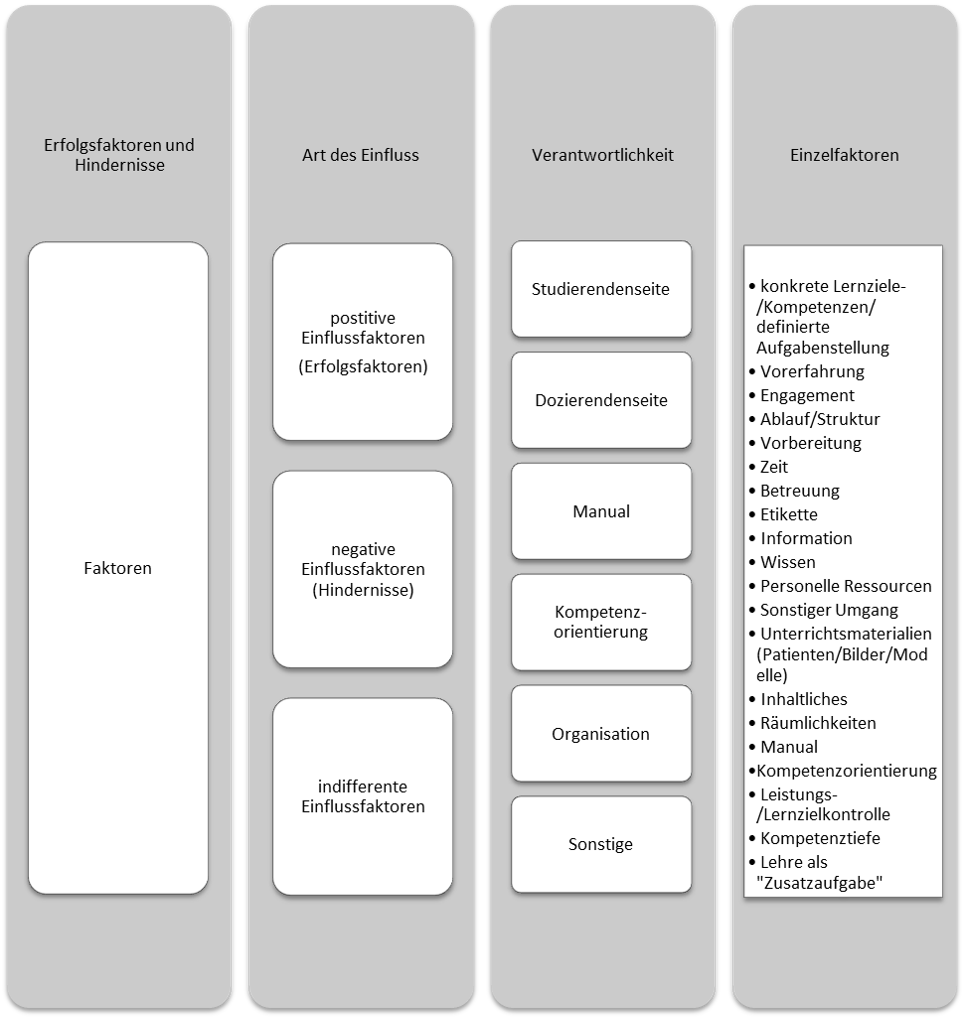

Supplement: Supplementary file 3 — Supplement 3: Übersicht über deduktiv und induktiv gebildete Kategorien [file 104_2024_2107_MOESM3_ESM.docx]

**
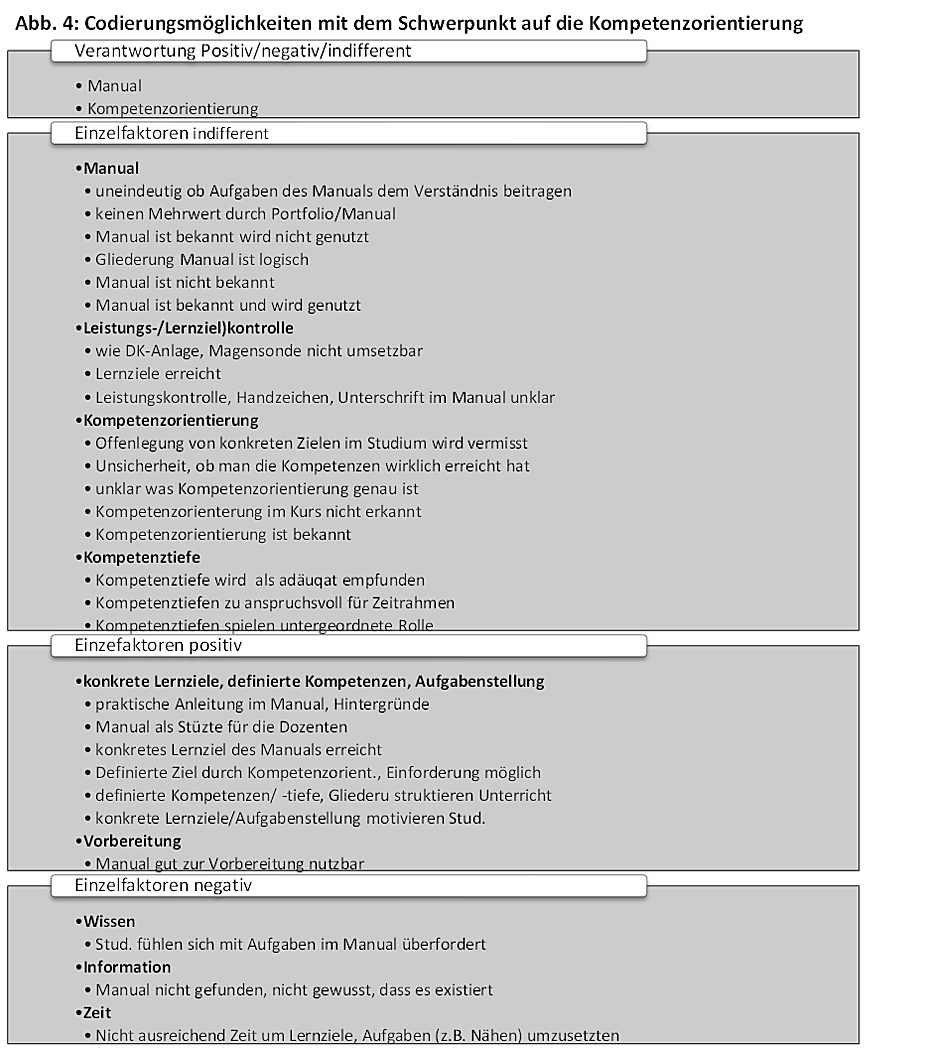
Supplement 4: Codierungsmöglichkeiten mit dem Schwerpunkt auf die Kompetenzorientierung**

Supplement: Supplementary file 4 — Supplement 4: Codierungsmöglichkeiten mit dem Schwerpunkt auf die Kompetenzorientierung [file 104_2024_2107_MOESM4_ESM.docx]
